# THE IMPACT OF COMMUNITY HEALTHCHOICES ON PERSON-CENTERED SERVICE PLANNING

**DOI:** 10.1093/geroni/igac059.566

**Published:** 2022-12-20

**Authors:** John Yauch

**Affiliations:** University of Pittsburgh, Pittsburgh, Pennsylvania, United States

## Abstract

Person-centered service planning (PCSP) is a required component of home and community-based services programs that operate under Medicaid waivers. CMS requires that states have processes in place to assure that participants and their family members (as desired and appropriate) are involved in decisions about their care. This mixed-method study combines interviews with participants and qualitative service coordinators regarding the PCSP process. Participant interviews using the Consumer Assessment of Health Providers Survey – Home and Community Based Services version found that the measure of “choice over services” improved slightly however, the “planning your care” measure was unchanged. Interviews with service coordinators found that the introduction of managed care had led to a system that was overly bureaucratic and focused on medical rather than social needs. This led to a perception that the service plans neglect aspects of daily living that are important for a good quality of life.

